# Lagrangian eddy kinetic energy of ocean mesoscale eddies and its application to the Northwestern Pacific

**DOI:** 10.1038/s41598-020-69503-z

**Published:** 2020-07-30

**Authors:** Mengrong Ding, Pengfei Lin, Hailong Liu, Aixue Hu, Chuanyu Liu

**Affiliations:** 1 State Key Laboratory of Numerical Modeling for Atmospheric Sciences and Geophysical Fluid Dynamics, Institute of Atmospheric Physics, Chinese Academy of Sciences, Beijing, China; 2 Climate & Global Dynamics Laboratory, National Center for Atmospheric Research, Boulder, CO USA; 3 College of Earth and Planetary Science, University of Chinese Academy of Sciences, Beijing, China; 4 Key Laboratory of Ocean Circulation and Waves, Institute of Oceanology, Chinese Academy of Sciences, Qingdao, China; 5 Laboratory for Ocean Dynamics and Climate, Qingdao Pilot National Laboratory for Marine Science and Technology, Qingdao, China; 6 Center for Ocean Mega-Science, Chinese Academy of Sciences, Qingdao, China

**Keywords:** Ocean sciences, Physical oceanography

## Abstract

Coherent oceanic mesoscale eddies with unique dynamical structures have great impacts on ocean transports and global climate. Eddy kinetic energy (EKE), derived from time-dependent circulation, is commonly used to study mesoscale eddies. However, there are three deficiencies of EKE when focusing on the analysis of coherent mesoscale eddies. Here, we propose a comprehensive concept—Lagrangian EKE (LEKE) as an additional metric which is a combination of gridded EKE calculated in Eulerian framework and tracked coherent mesoscale eddies in Lagrangian framework. Evidences suggest that LEKE can make up these deficiencies as an effective supplement. In this study, regional application over Northwestern Pacific Ocean is taken as an example. It clearly demonstrates that LEKE reveals more accurate and detailed characteristics of both cyclonic and anticyclonic eddies than EKE when coherent mesoscale eddies are the specific focus, such as the variation rates of kinetic energy during the eddy propagation, spatial–temporal differences of kinetic energy between cyclonic and anticyclonic eddies. Overall, using LEKE to analyze coherent mesoscale eddies gives the rise to understand the spatial–temporal contrasts between eddies with different polarities, and provides a new perspective to recognize the crucial role played by coherent mesoscale eddies in the ocean.

## Introduction

Ocean mesoscale eddies, with typical spatial scales on the order of tens to hundreds kilometers and temporal scales from weeks to months, are found almost everywhere in the global ocean^1,2^. Previous investigations suggest that collectively, mesoscale eddies are capable to exert significant impacts on large-scale ocean circulations^3,4^, basin-scale transports of mass, heat and bio-geo-properties^5,6^, global energy cycle^7,8^, and even synoptic weather patterns over remote domains^9,10^. Individually, the enclosed circulation of mesoscale eddies remains coherent (obvious self-organized structure) and allows the water mass trapped within their boundaries to transport the associated water properties (heat, momentum, etc.) over a long distance as these eddies move and evolve^11,12^. During their lifetimes, mesoscale eddies are affected and modulated by diapycnal mixing processes and upscale and (or) downscale energy cascades via interactions with the mean flow^13,14^. Owing to the distinct contrasts of their dynamical structures, mesoscale eddies are normally classified into cyclonic and anticyclonic eddies (hereafter CEs and AEs, respectively)^1,2^. It is manifest that CEs/AEs are reflected as closed contours with negative/positive sea surface height anomalies (SSHA). But the motions within either CEs or AEs are both characterized as roughly circular currents flowing around the eddy center^9^.

Because coherent mesoscale eddies capture almost 80% of the total kinetic energy (KE) in the ocean based on altimeter observations^15,16^, the surface gridded eddy KE (EKE), derived from the SSHA field based on the geostrophic relationship, is commonly used to analyze the mesoscale eddies and their variabilities^17–21^. However, the resulting EKE actually includes the KE of all mesoscale fluctuations—not only mesoscale eddies, but also meanders, fronts, waves and so on^16^. Statistical studies have explicitly suggested that mesoscale eddies should be treated separately from other mesoscale motions due to their coherent features with closed circulation and rotating structures^22^. Therefore, EKE is not an appropriate metric to characterize the KE of coherent mesoscale eddies. Generally, the signals of coherent mesoscale eddies could be obtained by applying spatial and/or temporal filters onto the SSHA or EKE field^23–25^. To properly do this, the typical spatial–temporal scales of mesoscale eddies (i.e., mean eddy size and lifetime) need to be identified in advance^25^. Since these scales are subject to specific geographical domain^25^, it would be difficult to apply a universal scale in the analysis focusing on coherent mesoscale eddies in the basin-wide or global ocean. Moreover, coherent mesoscale eddies are tracked in Lagrangian framework while the EKE is commonly calculated on the fixed grid boxes in Eulerian framework. The nonstationary features of coherent mesoscale eddies make it impossible to unveil the variations of KE during the period of eddy propagation by directly using the EKE alone. Furthermore, considering the substantial differences of effects on property transports^5,6^ and ocean mixing^14,26,27^ between AEs and CEs, it becomes essential for us to distinguish the KE of CEs and AEs in order to better understand their dynamical contrasts. Given all above, it calls for another metric to make up these deficiencies of EKE when focusing on coherent mesoscale eddies.

**Figure 1:**
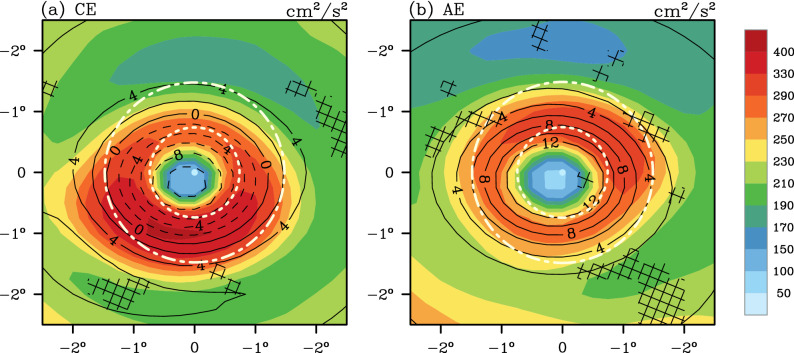
(**a**) Mean composite maps of eddy kinetic energy (EKE, shading, units: cm^2^/s^2^) and sea surface height anomalies (SSHA, contours, units: cm) for cyclonic eddies (CEs) over the Northwestern Pacific ocean. (**b**) The same with (**b**) but for anticyclonic eddies (AEs). In (**a**,**b**) the coordinate is built on the eddy center. The SSHA contour interval is 2 cm. The dashed lines (solid lines) represent negative (positive) values. The white dots represent the eddy center and the inner white circle denotes the average radii, while the outer white circle denotes the effective radii. Values in areas without hatched lines are statistically significant at the 95% confidence level, based on the *t*-test. The composite map (**a**)/(**b**) are averaged from 10,093 CEs/9,551 AEs tracked in the Northwestern Pacific ocean during the 1993–2016. The figure was made using NCAR Command Language 6.4.0 (https://www.ncl.ucar.edu/).

Before defining the additional metric for the KE of coherent mesoscale eddies, it is necessary to provide a basic interpretation collectively for the EKE distribution within the composite CEs and AEs (Fig. 1a,b). It is apparent that the EKE distribution within either CEs or AEs has a near symmetric ring structure. The eddy center (defined as the location with local extreme of SSHA, the white dot in Fig. 1a,b) is characterized as a relatively lower EKE with higher EKE in the surrounding (Fig. 1a,b). On average, the EKE at the eddy center (< 50 cm^2^/s^2^) is at least four times smaller than its vicinity (> 200 cm^2^/s^2^). Therefore, we add the concept of impacting area of each eddy as a circular shape based on its speed-based radius.

Apart from the impacting area, mesoscale eddies also feature discernable propagation and evolution during their lifetimes^28,29^. Coherent mesoscale eddies propagate at a speed nearly equal to the local phase speed of long baroclinic Rossby waves during their lifetimes^1,2^. During the propagation, these eddies evolve through three stages: growth, mature and decay^29^. In these three stages, mesoscale eddies behave differently: weak but changing rapidly in the growth and decay stages, while strong and stable in the mature stage^29^. These typical features of eddy propagation and evolution suggest that it is necessary to employ a coordinate moving with the mesoscale eddies in order to directly display the energetic evolution of each individual eddy.

Because of the distinct spatial–temporal variabilities and climatic impacts between CEs and AEs^5,30–32^, it is essential to analyze their KE separately. However, the usage of the gridded EKE is incapable of directly distinguishing the KE of eddies with different polarities. Although some studies attempted to isolate the KE of CEs from that of AEs based on the signs of corresponding SSHA^25^, the problem behind this attempt is that a negative (positive) SSHA does not always represent a CE (an AE) and cannot provide the factual information about the mesoscale coherent structures. Therefore, to probe into the differences between CEs and AEs, it is important to take the eddy polarity into account when defining the KE of coherent mesoscale eddies.

As the deficiencies of EKE outlined above, it calls for an additional metric, which collects and merges all information got from tracked coherent mesoscale eddies (impacting areas based on eddy size, eddy trajectories, eddy polarities), to give more accurately analysis of coherent mesoscale eddies. Although similar treatment has been put forward by some existing literatures^2,33–35^, many regarding issues still remain unknown. For example, are there inconsistencies of spatial–temporal variabilities between the gridded EKE and the KE owned by coherent mesoscale eddies? How the significant differences of the KE between CEs and AEs being illustrated by the geographical distribution? Will the geographical variation of EKE be influenced by the KE of mesoscale eddies at different stages? These issues have not been fully understood and remain challenging to us. It will significantly contribute to our knowledge on the mesoscale eddies’ role in the ocean by further examination of the KE focusing on coherent mesoscale eddies with different polarities at different stages.

For all the above reasons, the goal of this study is to propose a pertinent metric and to promote its usage in the researches on coherent mesoscale eddies. The metric is named as the Lagrangian EKE (LEKE), which is a combination of the gridded EKE in Eulerian framework and the tracked mesoscale eddies in Lagrangian framework. It can be seen as a supplement to the traditional EKE when the investigation focuses on coherent mesoscale eddies. Our results will show evidences of which LEKE is more accurate in measuring the KE of coherent mesoscale eddies with cyclonic and anticyclonic polarities. Along with the geographical distribution of LEKE of mesoscale eddies with different polarities at different evolutional stages, the spatial–temporal contrasts between the CEs and AEs will be displayed and analyzed. To provide a good illustration of the usage of LEKE, the Northwestern Pacific is chosen as an example.

## Results

**Figure 2:**
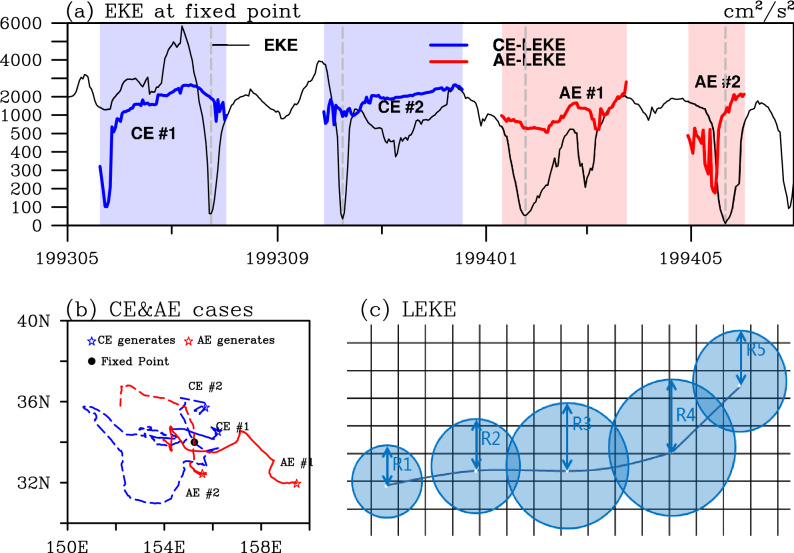
(**a**) Time series of daily EKE (units: cm^2^/s^2^) at the fixed point during 1 May 1993 to 30 June 1994. The light blue/red shadings show the impact stages when the fixed point is in the impacting area of CEs/AEs. The thick blue/red lines indicate the Lagrangian EKE (LEKE, units: cm^2^/s^2^) of the corresponding CEs/AEs during the impact stages. The vertical dashed lines denote the day when the fixed point is overlaid by the eddy center. (**b**) Trajectories of the CEs and AEs with blue and red lines, respectively. The locations where the eddies originally generated are marked with star symbols while the fixed point is marked with a black dot symbol. (**c**) Schematic diagram of LEKE calculation. The white dots represent the eddy center and the shading circles represent the impacting area S. The figure was made using NCAR Command Language 6.4.0 (https://www.ncl.ucar.edu/).

As mentioned earlier, the commonly used EKE is a mixture of the KE of different meso- (to smaller) scale fluctuations, such as mesoscale eddies, fronts, meanders and so on. Here, the LEKE we proposed is defined as the weighted area-averaged EKE over each eddy’s impacting area. We highlight the difference between EKE and LEKE by taking a random fixed point near the Kuroshio Extension Jet (the black dot in Fig. 2b) as an example. During the period of May 1st, 1993 to June 30th 1994, four eddies passed by this point, including two CEs and two AEs. As shown in Fig. 2a, the daily EKE during the whole period varies over time drastically, ranging from as low as 10 cm^2^/s^2^ to around 6,000 cm^2^/s^2^. The mean value of EKE over the time span (425 days) is 1,421 cm^2^/s^2^. The entire period for a given fixed point is divided into two types: the periods with (impacting periods, colored shadings) and without (no colored shadings) the influences of coherent mesoscale eddies. The impacting periods are periods when this fixed point is within the impacting area of a CE or an AE. The EKE over each impacting period varies within a large range in its own way. The magnitude of EKE during the periods without the influences of any mesoscale eddy can also reach up to over 2000 cm^2^/s^2^ (for instance, in the mid-May 1993). Although the EKE values in or out of the impacting periods can be comparable to each other, the impacting periods take a larger percentage of the total time span (67.8%). This indicates that the coherent mesoscale eddies contribute more to the EKE than other mesoscale fluctuations. Furthermore, the EKE calculated at the fixed point can only provide discrete value of the KE of the eddies passing by, and the time evolution of these coherent mesoscale eddies is unable to be identified. With this in mind, a coordinate moving with the mesoscale eddies is needed and the information of tracked eddies should be merged into the definition of LEKE (Fig. 2c). By this way, the KE of coherent mesoscale eddies, namely LEKE, can be isolated from the gridded EKE. In the following paragraphs, the three advantages of LEKE in analyzing coherent mesoscale eddies are further presented.

The first advantage of LEKE over EKE is that the former can measure the KE of coherent mesoscale eddies more accurately. Due to the symmetric ring structure of EKE within mesoscale eddies, when a fixed point is overlaid by an eddy center, the EKE at that fixed point shows a much smaller value than the LEKE of the identified eddy (Table 1). As shown in Table 1, it is obvious that LEKE of mesoscale eddies can be much larger (over 10–100 times in the four cases we picked) than the EKE at a given fixed point. This means that the EKE at a fixed point cannot accurately represent the KE of a given eddy as a whole, but only a certain part of an eddy. If the EKE averaged in a small box is used to represent the KE of the mesoscale eddies, the KE of these eddies could be significantly underestimated. To represent the KE of mesoscale eddies in a region, the weighted area-averaged EKE over a specific region (such as a box of 2° × 2°^36^ or up) has been employed in many previous studies^17–21^. Based on the above illustration, the resulting EKE in these analyses may not be appropriate in representing coherent mesoscale eddies. Therefore, the calculation of LEKE proposed here takes the entire impacting area of mesoscale eddies as an integral to give a more accurate evaluation of the KE of coherent mesoscale eddies.

**Table 1:** The eddy kinetic energy (EKE, units: cm^2^/s^2^) at fixed point and the Lagrangian EKE (LEKE, units: cm^2^/s^2^) of the mesoscale eddy when the fixed point (the black dot in Fig. 1a) is overlaid by the eddy center.

No	LEKE of the mesoscale eddy	EKE at the fixed point
#1 CE	1906.3	63.8
#2 CE	937.7	36.0
#1 AE	602.5	54.2
#2 AE	1,110.4	11.2

**Figure 3:**
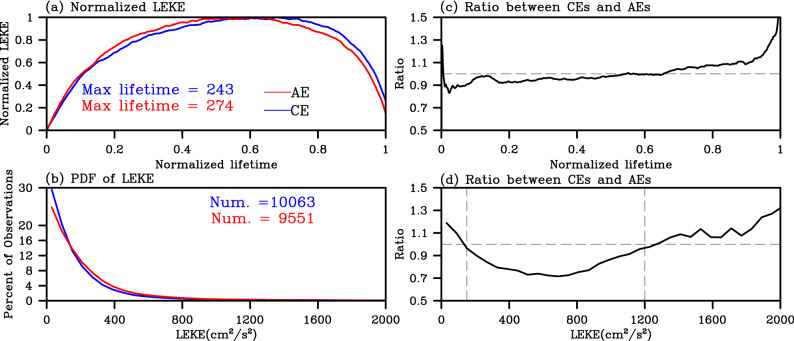
(**a**) Mean evolutions of normalized CE-LEKE (blue line) and AE-LEKE (red line) over the Northwestern Pacific Ocean. The maximum lifetimes of eddies are labeled in the panel (blue for CEs and red for AEs). (**b**) Histograms of CE-LEKE (blue line) and AE-LEKE (red line) over the Northwestern Pacific Ocean. The numbers of trajectories are labeled in the panel (blue for CEs and red for AEs). (**c**,**d**) The ratio of the value for CE-LEKE to that of AE-LEKE from Fig. 3a-b, respectively. The dashed line denotes the ratio value 1. The figure was made using NCAR Command Language 6.4.0 (https://www.ncl.ucar.edu/).

The second advantage is that LEKE can be used to describe the time evolution of KE of coherent mesoscale eddies in either group or individual analysis. For each tracked mesoscale eddy, the LEKE from generation to dissipation can be recorded and analyzed separately. The LEKE can be indicative of its intensity of eddies and the variation during the eddy lifetime. For an individual mesoscale eddy, it becomes easy to depict where and when it is gaining or losing energy based on the variation of LEKE during its lifetime. Taken as a group, the common evolution process of coherent mesoscale eddies from growth, mature to decay can be obtained by averaging over all the mesoscale eddies identified in a relative large domain (Fig. 3a, the Northwestern Pacific Ocean is used as an example). The evolution process is able to be used to compare with those of other eddy kinematic properties, such as the radius, amplitude and intensity^33,34^. Here, the LEKE of each eddy is normalized by its maximum difference value during its lifetime in order to compare more objectively the LEKE from different eddies since the LEKE of different eddies varies in a large range from O(10) to O(10^3^) (Fig. 3b). There are some interesting features can be easily seen after the normalization. For example, during the normalized lifetimes, coherent mesoscale eddies take over two fifths of their lifetimes to enter into the stable stage while it takes only one fifths to decay (Fig. 3a). This suggests that the KE of coherent mesoscale eddies is much faster to be dissipated than to be gained. Such information is helpful for us to gain a better understanding on the eddy energy sources and sinks in different domains if we further take all the individuals as a collection.

**Figure 4:**
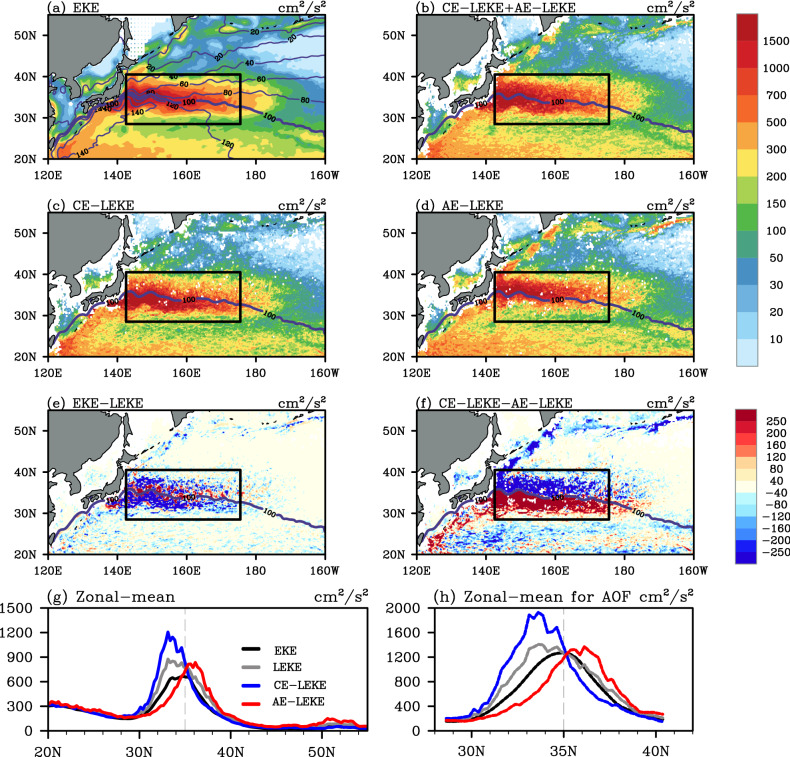
Mean state and the latitudinal distribution of KE of eddy activity in the Northwestern Pacific Ocean during 1993–2016. (**a**) Mean EKE (shading, units: cm^2^/s^2^) and absolute dynamic topography (ADT, contours, units: cm). (**b**) Mean LEKE (units: cm^2^/s^2^) for all identified eddies. (**c**) Mean CE-LEKE. (**d**) Mean AE-LEKE. The ADT contour interval is 20 cm in (**a**) and the 100-cm contour of mean ADT is marked in (**a**–**d**). The black rectangles denote the Kuroshio Extension region for Table 2. (**e**) The difference between (**a**) and (**b**). (**f**) The difference between (**c**) and (**d**). (**g**) The latitudinal distribution of EKE (black line), LEKE of all eddies (grey line), CE-LEKE (blue line) and AE-LEKE (red line) for the Northwestern Pacific Ocean, with the vertical dashed lines denote the latitude of 35° N. (**h**) The same as (**e**) but for the Kuroshio Extension region. The figure was made using NCAR Command Language 6.4.0 (https://www.ncl.ucar.edu/).

The third advantage of the LEKE is its capability to differentiate the KE of CEs and AEs directly. Since the CEs and AEs are usually identified and tracked separately, the LEKE can be calculated for CEs and AEs, respectively. Subsequently, the differences between these two types of eddies can be analyzed. The first difference is shown in the evolution process of LEKE (Fig. 3a). The AE-LEKE grows faster than CE-LEKE during the first three-fifths of the normalized eddy lifetime (Fig. 3c, the Northwestern Pacific Ocean is used as an example). For the last two-fifths, the AE-LEKE decays slower than CE-LEKE. This can partially explain why AEs have longer lifetimes than CEs, as previous studies mentioned^37^. The second difference is displayed from probability density function of the LEKE (Fig. 3b). There are more AEs than CEs with LEKE between 150–1,200 cm^2^/s^2^ (Fig. 3b,d). According to the statistics, the majority (99%) of the CEs and AEs has their LEKE less than 600 and 700 cm^2^/s^2^, respectively. This suggests that CE-LEKE over this domain has relatively wider range than AE-LEKE. The third difference exists in the spatial–temporal variabilities of CEs and AEs. Taking the spatial distribution as example, the magnitude of mean AE-LEKE is stronger in the coastal seas of Northwestern Pacific Ocean than that of CE-LEKE (Fig. 4c,d,f). A more thorough description of this difference is given in the following paragraphs. It is worth to note that the degrees of above differences between CE-LEKE and AE-LEKE may vary in other geographical domains. Nevertheless, all these differences confirm the necessity and significance of using LEKE to analyze coherent mesoscale eddies as an additional metric for EKE.

Because of its easy accessibility^17–21^, the gridded EKE has been broadly used in the previous analysis of KE of coherent mesoscale eddies. The question naturally being asked is whether LEKE capture the same spatial variation with EKE. To test this issue, LEKE of all detected eddies is re-gridded back into the same resolution as EKE (daily, 1/4°). After averaging the daily LEKE filed over all time steps, the climatological geographical distribution of the EKE and LEKE can be compared. Here, the Northwestern Pacific Ocean is continuously used as an example. As shown in Fig. 4a, the large values of the climatological mean EKE are found in the vicinity of Kuroshio Extension Jet (defined as the 100-cm contour absolute dynamical topography^17,38^). The distribution of these large values is roughly symmetric along the Kuroshio Extension Jet (black lines in Fig. 4g,h). The climatologically geographical distribution of the LEKE of all identified mesoscale eddies (Fig. 4b) shares a similar spatial pattern with that of EKE (with a spatial correlation coefficient up to 0.86) along with some minor differences. This further confirms that coherent mesoscale eddies are the main contributors to the oceanic KE.

By comparing the spatial distributions of LEKE or EKE with CE-LEKE and AE-LEKE respectively, the different contributions of CE-LEKE and AE-LEKE to the total LEKE, as well as to the EKE, can be presented. The spatial correlation coefficient between LEKE and CE-LEKE is 0.92 which is larger than that with AE-LEKE (0.76). The main difference between CE-LEKE and AE-LEKE locates alongside the Kuroshio Extension Jet (denoted by the thickened 100-cm contour of absolute dynamical topography in Fig. 4) and the coastal areas of Northwestern Pacific (Fig. 4f). The AE-LEKE is significantly smaller/larger than CE-LEKE in the southern/northern side of the Kuroshio Extension Jet (Fig. 4e). Over the marginal seas from northeast of Japan to south of Aleutian Islands, the AE-LEKE is much stronger than the CE-LEKE and the difference can be as large as 300–400 cm^2^/s^2^. This is because the identified AEs over this region have longer lifetime than CEs^2^. A further division of the Northwestern Pacific Ocean shows that AE-LEKE average over the north of Kuroshio Extension Jet (locating around 35°N) has a larger spatial correlation coefficient with LEKE than CE-LEKE (0.95 versus 0.89). Over the region south of 35°N, it is the opposite situation (0.69 versus 0.95). Taken the Kuroshio Extension region (the black rectangles in Fig. 4a–d) as a whole, the LEKE differences between CEs and AEs are even more obvious: the maximal value of zonal mean of CE-LEKE is at the latitude of 33°N while that for AEs is located at 36.5°N (Fig. 4h). Therefore, it is evident that the LEKE can complement the deficiencies of EKE in representing the mesoscale eddy field with different polarities.

Next, we shift to the temporal variability shown by LEKE. The weighted area-averaged results of EKE and LEKE over the Kuroshio Extension region during 1993–2016 are compared (Table 2). The area-averaged LEKE is larger (8%) than that of the EKE. This implies that coherent mesoscale eddies are in general stronger than other mesoscale fluctuations (Fig. 4e). With a much larger standard deviation (STD), LEKE displays a larger variation than EKE. So it confirms that coherent mesoscale eddies are contributing to a larger variation of EKE than other mesoscale motions. Furthermore, there is no statistically significant trend for EKE during this period in this region, while the trend of LEKE is 1.99 cm^2^/s^2^/year which is statistically significant at 95% significance level. Meanwhile, the trends of CE-LEKE and AE-LEKE are also significant and their values are 1.79 cm^2^/s^2^/year and 1.83 cm^2^/s^2^/year, respectively. This infers that other mesoscale motions may have a decreasing trend over this 24-years period which offsets the increasing trend of coherent mesoscale eddies. This results in a non-significant trend in EKE. The stronger trend of coherent mesoscale eddies may be related with the changes in ocean stratification^39^. With a warmer climate, the overall surface ocean stratification strengthens and this stronger stratification will prevent a deep penetration of these eddies. Even with an unchanged wind stress field working on such a thinner ocean layer, mesoscale eddies spin faster with a higher rotational velocity and stronger intensity. Thus the coherent mesoscale eddies demonstrate a significant long term trend. Further work is needed to study the resulted trend, but beyond the scope of this study.

**Table 2:** Mean value, standard deviation and trend (/year) with p value in the parentheses for the area-averaged EKE (units: cm^2^/s^2^), LEKE (units: cm^2^/s^2^) and eddy amplitude (units: cm) over the Kuroshio Extension region (black rectangle in Fig. 1a).

	Mean value	Standard deviation	Trend
EKE	603.67	83.89	0.33 (0.65)
LEKE	651.83	119.93	**1.99 (0.00)**
CE-LEKE	716.05	177.40	**1.79 (0.01)**
AE-LEKE	583.7	101.0	**1.83 (0.01)**
CE-amplitude	14.93	1.76	− 0.02 (0.98)
AE-amplitude	14.20	1.65	0.00 (1.00)

The bold ones represent the trend that has passed the 95% significance test.

The differences on temporal scale between the CEs and AEs are also apparent over the Kuroshio Extension region. The CEs is stronger than AEs with an average value of 716.1 cm^2^/s^2^ and 583.7 cm^2^/s^2^, respectively. The variance of CE-LEKE, measured as STD, is also larger than that for AE-LEKE which suggests that the CEs over this region show a more significant variability than the AEs. Similar to the LEKE of all identified eddies, both CE-LEKE and AE-LEKE increase significantly (at 99% level) over 1993–2016. As shown in Table 2, if EKE and eddy amplitudes, are used in examining coherent mesoscale eddies, these increasing trends of coherent mesoscale eddies will be undetected. Thus, the usage of LEKE is fundamental to explore the temporal contrasts between CEs and AEs.

In summary, the additional metric LEKE gives a more accurate and detailed description of the characteristics of coherent mesoscale eddies than EKE. The usage of LEKE helps to put great emphasis on the crucial role played by coherent mesoscale eddies in the ocean. Furthermore, LEKE can provide a more thorough understanding of the spatial–temporal contrasts between CEs and AEs. Certainly, the inconsistencies between EKE and LEKE, CE-LEKE and AE-LEKE are worthy of further investigation. There are some speculations given in the next section.

## Conclusions and discussion

By combining EKE calculated in Eulerian framework with tracked eddies in Lagrangian framework, LEKE is proposed to measure the KE of coherent mesoscale eddies. LEKE can reveal more accurate and detailed characteristics of coherent mesoscale eddies than EKE. This is related with the three deficiencies of EKE when focusing especially on mesoscale eddies with coherent rotating structures. Firstly, EKE averaged over a given region is a mixture of the KE of all mesoscale motions. Secondly, EKE fails to display time evolution of mesoscale eddies. Last and most important, EKE is incapable of distinguishing the KE of CEs and AEs. By taking the Kuroshio Extension region as an example, the spatial–temporal variabilities of EKE and LEKE are compared. The roles of coherent mesoscale eddies are highlighted with the usage of LEKE. More information, such as the relative contribution of CEs and AEs in south/north of Kuroshio Extension Jet, the larger temporal variability with a significant increasing trend and so on are all provided to a deep understanding of coherent mesoscale eddies over this domain. It is evident that LEKE as an addition metric to analyze coherent mesoscale can draw further attention to their unique roles in the ocean.

With regard to the inconsistency between EKE and LEKE, two major reasons are speculated. Firstly, LEKE includes only the KE from coherent mesoscale eddies, while the gridded EKE calculated in Eulerian framework represents the KE from coherent mesoscale eddies and all other mesoscale fluctuations. Secondly, LEKE treats EKE within the impacting area of an eddy as a whole, but fixed gridded EKE only represent the KE at a given grid box. However, for either a CE or an AE, it typically has a symmetric ring structure with a low EKE value at the center and larger EKE value in the vicinity. So the LEKE proposed here is defined as the weighted area-average EKE within the impacting area of a given eddy. One thing to note is that the eddy impacting area in our study is calculated using the speed-based radius of each mesoscale eddy by assuming a regular circular structure of these eddies. Other definitions^2^ of eddy size (e-folding radius and effective radius) are also checked and analyzed (figures not shown). The results are very similar to the LEKE shown in this study except some differences in magnitude. The LEKE differences among different choices of eddy radius become more significant in regions with strong background circulation. But the choice of eddy radius in defining the LEKE does not affect our conclusions provided here. When LEKE is re-gridded back to the same horizontal resolution as EKE, the LEKE of an eddy is assigned to the location of this eddy’s center, which also leads to certain spatial differences between EKE and LEKE. Undoubtedly, other eddy properties, such as the local circulation, the seasonal variability of Kuroshio Extension system or else (figures not shown), may have also contributed to these differences. Further research is needed in order to better understand these differences, but beyond the scope of this study.

The difference between CEs and AEs is also a point of interest. This difference might be associated with the background mean flow. The interactions between the mesoscale eddies and mean flow insert considerable influences onto the eddy genesis and propagation. Coherent CEs and AEs in different ranges of lifetimes have distinct different spatial distribution in the Kuroshio Extension region^37^. This is because that the Kuroshio Extension Jet induces two different eddy generation mechanisms. In other words, the background mean flow can lead to the difference between the CEs and AEs at the beginning of eddies’ lifetime. As eddies propagate away from their genesis region, the interaction with the background circulation could also slow down or accelerate the decaying processes of the CEs and (or) AEs^40^. Work will also be needed to deepen our knowledge of differences between CEs and AEs.

As mentioned above, LEKE can be used to track the evolution of the KE of each individual mesoscale eddy during its lifetime. As a result, the geographical distribution of LEKE at different stages can be obtained. Although the spatial patterns of the gridded LEKE in three different life stages share similar spatial distributions in the Northwestern Pacific Ocean, the magnitude of LEKE is the largest during eddy’s mature stage (Fig. S1a–c). Comparing the growth stage with decay stage (Fig. S1e), LEKE is larger in the latter than in the former, especially in the regions south of 42°N. The largest difference occurs at 33°N, which is up to 40 cm^2^/s^2^. Further analysis indicates that CEs contribute more to the variations of LEKE at decay stage between 30° and 35°N (Fig. S2). Combining these results, it gives a conclusion that the eddy termination occurs more frequent over the Kuroshio Extension region and CEs devotes more. This information provides a new perspective to recognize the crucial role played by coherent mesoscale eddies in the ocean.

LEKE could also provide a potential linkage for the further understanding of the energy cascade at both directions. Recent results have shown an enhanced mixing due to wind-driven internal waves where the EKE is relatively large^17^. Moreover, this mixing is more powerful within the AEs than that within the CEs in the region of 30°–45°N^27^. The spatial difference between CEs and AEs shields more lights on the roles played by CEs and AEs in ocean mixing. For instance, the mixing is found to be enhanced in the band of 20°–22°N, which is due to the effect of AEs^27^. It may be corresponded with larger AE-LEKE (over 40%) than that CE-LEKE at the longitude of 143°E (Fig. S3). These evidences do produce practical significance on the further understanding of inverse energy cascade.

Although LEKE is evidenced to be helpful in the targeted analysis of coherent mesoscale eddies, there is still something unclear about the calculation of LEKE which needs to be mentioned. In this study, the entire impacting area (defined by the speed-based eddy radius) as an integral is employed into the calculation of LEKE. This is based on the fact that there is always a strongly self-organized structure within a given coherent mesoscale eddy. Thus the derived LEKE is significantly related to the variation of eddy radius during eddy lifetime (figures not shown). As for the obtained eddy radius from the eddy trajectories dataset, it is highly sensitive to the definition of eddy boundary (the eddy identification-tracking method) and input data^2^. In this study, the global mesoscale eddy trajectory atlas product version 1.0 distributed by AVISO+ has been used. Analyses have pointed out that the eddy boundary has large uncertainties in different identification and tracking methods^2,41–43^. The eddy identification and tracking algorithm used to derive this eddy trajectory dataset did not take the eddy merging and splitting events into account so some identified eddies have certain multinuclear structures^2,22,41^. This may lead to the large fluctuation of eddy radius and then that of the derived LEKE during a short time span, such as AE # 2 shown by the Fig. 2a. The novel eddy identification algorithms^42,43^ which can detect mononuclear eddies may obtain a more accurate LEKE with a much smoother evolution of LEKE during eddy lifetime. Meanwhile, coherent mesoscale eddies are usually not exactly circular, so errors could rise from any of the abovementioned choices of eddy radius since they all assume a circular eddy structure. Thus, the eddy identification and tracking algorithm may be one factor which will affect the LEKE calculation. Besides this, the input data for deriving the eddy trajectory dataset can also affect the eddy features, especially for eddy boundary. In this eddy trajectory dataset, the DUCAS “two-satellite” daily SSHA product with a spatial resolution of 1/4° has been used to identify mesoscale eddies. This input data with an effective resolution coarser than 1/4° will result in the mis-location of the identified eddy centroids and further lead to the misinterpretation of eddy radius since this eddy trajectory dataset is essentially derived with a SSH-based automated eddy identification method. Therefore, addition works are needed in order to more comprehensively determine which way to define eddy impacting area is better.

Another caveat worth to mention is that ocean eddies have three-dimensional structures while LEKE here is calculated only based on the surface information from eddy trajectories. To better understand the eddy dynamics, the definition of LEKE should be extended to the three-dimensional structure obtained from high-resolution ocean observations or from eddy-resolving numerical models.

## Methods

In this study, the SSHA data is used to calculate the EKE: $\text{EKE}=1/2\left({U}_{g}^{{^{\prime}}2}+{V}_{g}^{{^{\prime}}2}\right)$, where ${U}_{g}^{^{\prime}}=-g/f(\partial {h}^{^{\prime}}/\partial y)$ and ${V}_{g}^{^{\prime}}=g/f(\partial {h}^{^{\prime}}/\partial x)$ are the zonal and meridional surface geostrophic velocity anomalies, respectively, with $f$ being the Coriolis parameter, $g$ being the gravitational constant, ${h}^{^{\prime}}$ being the sea surface height anomalies (SSHA). The data is distributed by the Archiving, Validation and Interpretation of Satellite Oceanographic Data (AVISO, https://www.aviso.altimetry.fr/). Its spatial and temporal resolutions are 1/4° and daily, respectively. In addition to the SSHA data, we also adopt the absolute dynamic topography (ADT) from AVISO, which is with the same resolutions as the SSHA data.

The global mesoscale eddy trajectory atlas product version 1.0 is used in this study to get the tracking information of each mesoscale eddy. This dataset is produced by the Segment Sol multimissions d’ALTimétrie, d’Orbitographie et de localization Precise (SSALTO)/ Data Unification and Altimeter Combination System (DUACS) multi-mission altimeter data and distributed by AVISO in collaboration with the Oregon State University. The eddy identification and tracking methods used for this product are detailed described^2,41^. Usually a limit of eddy lifetime (28 days in general) is applied to ensure the certain existence of detected mesoscale eddy because of the possible error of altimeter data. Concurrently, the minimum limit of lifetime ensures the identified mesoscale eddies as coherent structures^1–2,22^. Therefore, we mainly focus on KE of eddies with the minimum lifetime of 28 days in this study. Other properties, such as the time and locations when and where eddies are detected, the corresponding eddy radius, amplitude are all included in this dataset. The comprehensive information about this dataset can be found on the AVISO website (https://www.aviso.altimetry.fr/).

### Lagrangian eddy kinetic energy

In this study, the LEKE is proposed to measure the KE of each individual mesoscale eddy. The LEKE of a given mesoscale eddy is obtained by the following four steps.

Step one, the mesoscale eddies are identified and tracked if the SSHA or other high-resolution observational datasets are provided. In our study, the eddy trajectories dataset released by AVISO are directly used.

Step two, the surface EKE field is derived from the gridded SSHA data based on the geostrophic relationship.

Step three, the impacting area S of this identified eddy is determined in the EKE map of the corresponding time step. In this step, the speed-based radius (the radius of the circle that has the same area as the region within the closed contour of SSH with maximum average speed^1,2^) is considered and merged into the definition. S in this study denotes the covering circular shape within the distance of one speed-based radius from the eddy center. As the size of each individual eddy varies during its lifetime, the impacting area S for each eddy at the given time step also change.

Step four, the LEKE of each eddy is defined as the weighted area-averaged EKE over the eddy impacting area S, ${\text{LEKE}} = \iint {\text{EKE}}\cdot{\upomega}_{\text{x}}\cdot{\upomega}_{\text{y}}{\text{dS}}/ \iint {\upomega}_{\text{x}}\cdot{\upomega}_{\text{y}}{\text{dS}}$, with ${\upomega}_{\text{x}}$ and ${\upomega}_{\text{y}}$ being the zonal and meridional weights subject to the longitude and latitude and S being the impacting area of the given mesoscale eddy. To display the time evolution of LEKE, the LEKE is calculated in every identified time step at the location where the eddy is identified. In addition, the CE-LEKE and AE-LEKE are calculated separately so the individual contribution of CEs and AEs to the oceanic KE can be assessed.

Finally, we obtain the LEKE of the given eddy at the given time step. The four-steps procedure is repeated to all other qualified coherent mesoscale eddies at every identified time steps and then the LEKE dataset is obtained.

In order to simply exhibit the evolution process of each eddy, both its LEKE magnitude and lifetime are normalized, so that it has a maximum and minimum normalized LEKE, denoted as $\text{LEKE}_{(i,t)}^{^{\prime}}$, of 1 and 0, respectively and has a normalized lifetime of 1. Here the LEKE magnitude for each individual eddy is normalized by its maximum difference value during its lifetime, $\text{LEKE}_{(i,t)}^{^{\prime}}=\frac{{\text{LEKE}}_{(i,t)}-\min({LEKE}_{i})}{\max({\text{LEKE}}_{i})-\min({\text{LEKE}}_{i})}$.

In addition, to compare with the common gridded EKE, the LEKE of all the identified eddies (denoted as LEKE-occurrence) is gridded into daily and 1/4° grid boxes. During the gridded process, the value of LEKE is assigned to the eddy center as the KE of this eddy. Since the LEKE is calculated at every identified time step, the LEKE of coherent mesoscale eddies at different stages can be gridded separately. The LEKE of the eddies’ growth stage (the day eddy generates, denoted as LEKE-generation), decay stage (the day eddy terminates, denoted as LEKE-termination) and mature stage (the day eddy gets the largest amplitude, denoted as LEKE-largest) are all picked for each individual eddy and gridded into daily and 3° grid boxes (the grid boxes is lager in order to make sure each grid box has enough samples). Taking this into account, more implications can be acquired in the investigation of the geographical distribution of LEKE of coherent mesoscale eddies at different stages.

## Supplementary information

Supplementary Information 1.
