# Supplementary material for: Lagrangian eddy kinetic energy of ocean mesoscale eddies and its application to the Northwestern Pacific
Source: Sci Rep. 2020 Jul 30;10:12791. doi: 10.1038/s41598-020-69503-z (PMC7393132; doi:10.1038/s41598-020-69503-z)
Supplement: Supplementary file 1 — Supplementary Information 1. [file 41598_2020_69503_MOESM1_ESM.pdf]

Supplement of

Lagrangian Eddy Kinetic Energy of Ocean Mesoscale Eddies and its Application to the Northwestern Pacific

Mengrong Ding[1, 2, 3], Pengfei Lin[1, 3]*, Hailong Liu[1, 3]*, Aixue Hu[2], Chuanyu Liu[4, 5, 6]

[1]State Key Laboratory of Numerical Modeling for Atmospheric Sciences and Geophysical Fluid Dynamics, Institute of Atmospheric Physics, Chinese Academy of Sciences, Beijing, China

[2]Climate & Global Dynamics Laboratory, National Center for Atmospheric Research, Boulder, Colorado, USA

[3]College of Earth and Planetary Science, University of Chinese Academy of Sciences, Beijing, China

[4]Key Laboratory of Ocean Circulation and Waves, Institute of Oceanology, Chinese Academy of Sciences, Qingdao, China

[5]Laboratory for Ocean Dynamics and Climate, Qingdao Pilot National Laboratory for Marine Science and Technology, Qingdao, China,

[6]Center for Ocean Mega-Science, Chinese Academy of Sciences, Qingdao, China

Corresponding author: Pengfei Lin, Hailong Liu

Email: linpf@mail.iap.ac.cn, lhl@lasg.iap.ac.cn

Tel: 010-82995223

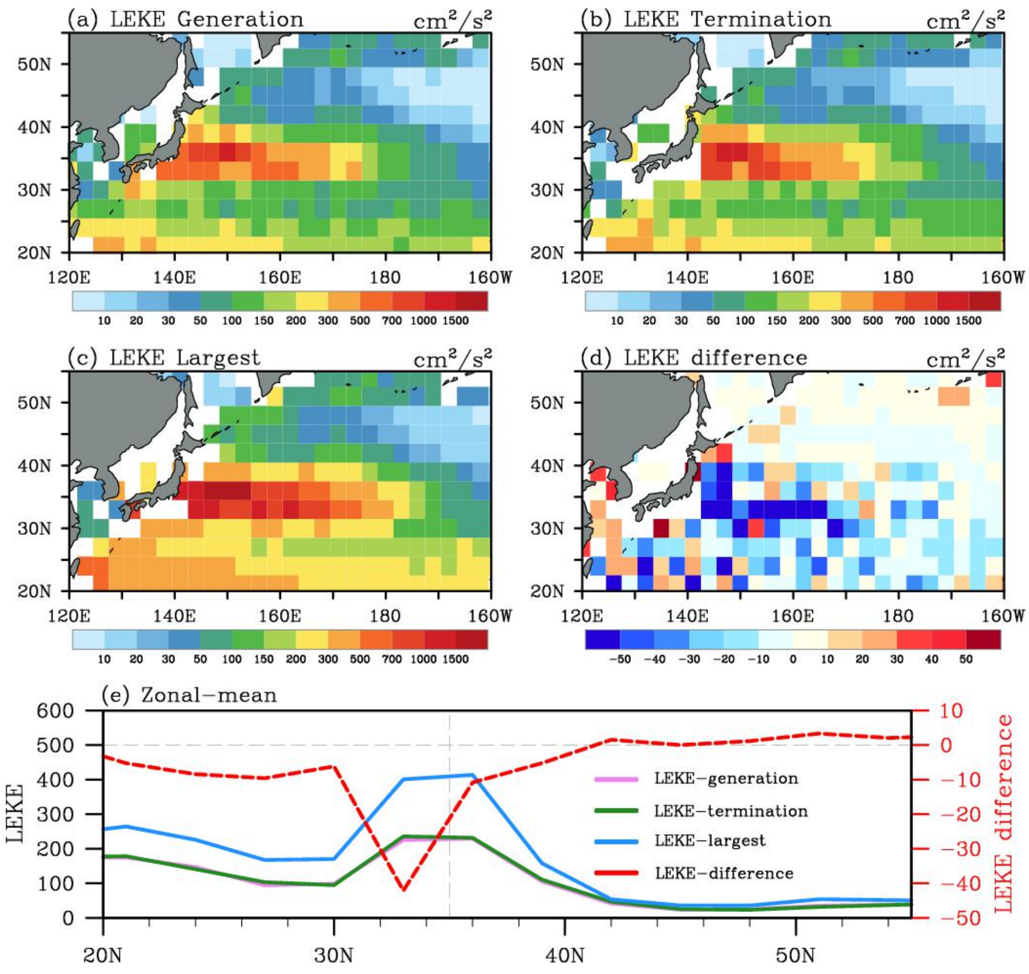

Fig. S1 Mean state and the latitudinal distribution of kinetic energy for eddy activity in the northwestern Pacific Ocean during 1993-2016. (a) Mean Langrangian eddy kinetic energy (LEKE)-generation (in shading, units: cm^2/s^2). (b) Mean LEKE-termination (units: cm^2/s^2). (c) Mean LEKE-largest. (d) Difference between the Fig. (a) and Fig. (b). (e) The latitudinal distribution of kinetic energy in (a-d). The latitudinal distribution of (d) corresponds with the right y-axis. The figure was made using NCAR Command Language 6.4.0 (http://www.ncl.ucar.edu/).

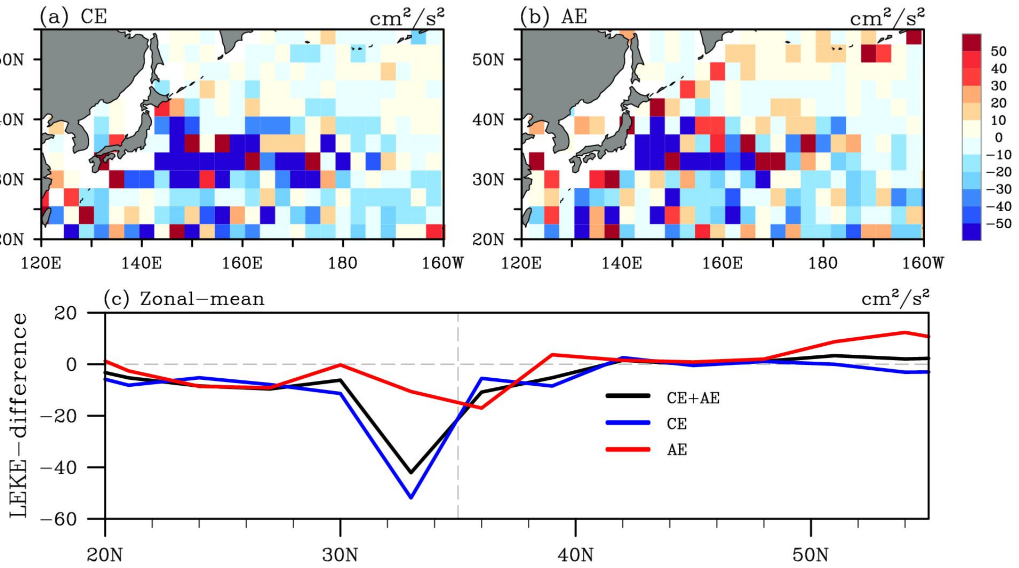

Fig. S2 Similar to the Fig. S1(d), but for (a) cyclonic eddies (CEs) and (b) anticyclonic eddies (AEs). (c) The latitudinal distribution of LEKE-difference for all identified eddies (black line), CEs (blue line) and AEs (red line). The figure was made using NCAR Command Language 6.4.0 (http://www.ncl.ucar.edu/).

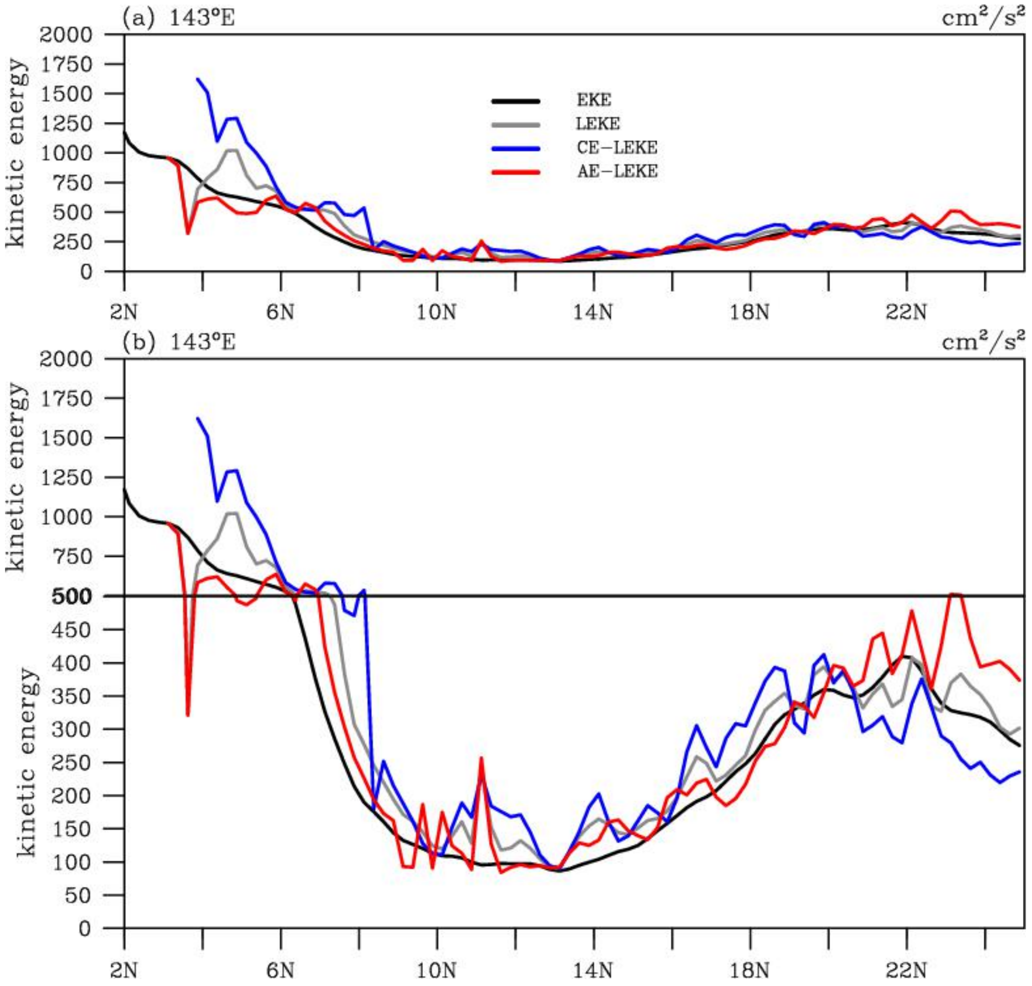

Fig. S3 Eddy kinetic energy (EKE, black line), LEKE for all identified eddies (grey line), CEs (blue line) and AEs (red line) at the longitude of 143ºE.
